# Presence of Mental Imagery Associated with Chronic Pelvic Pain: A Pilot Study

**DOI:** 10.1111/j.1526-4637.2011.01152.x

**Published:** 2011-06-13

**Authors:** Chantal Berna, Katy Vincent, Jane Moore, Irene Tracey, Guy M Goodwin, Emily A Holmes

**Affiliations:** * Department of Psychiatry, University of OxfordWarneford Hospital, Oxford; † Centre for Functional Magnetic Resonance Imaging of the Brain (FMRIB), Department of Clinical Neurology & Nuffield Department of Anaesthetics, University of OxfordOxford; ‡ Nuffield Department of Obstetrics and Gynaecology, University of Oxford, John Radcliffe HospitalOxford, UK

**Keywords:** Mental Imagery, Chronic Pain, Pelvic Pain, Cognition, Cognitive Behavioral Therapy

## Abstract

**Objective:**

To ascertain whether a small sample of patients with chronic pelvic pain experienced any pain-related cognitions in the form of mental images.

**Patients:**

Ten women with chronic pelvic pain consecutively referred from a tertiary referral center by the physicians in charge of their treatment.

**Outcome measures:**

An interview was used to determine the presence, emotional valence, content, and impact of cognitions about pain in the form of mental images and verbal thoughts. The Brief Pain Inventory (BPI), Pain Catastrophizing Scale (PCS), Spontaneous Use of Imagery Scale (SUIS), and Hospital Anxiety and Depression Scale (HADS) were completed.

**Results:**

In a population of patients with a prolonged duration of pain and high distress, all patients reported experiencing cognitions about pain in the form of mental images. For each patient, the most significant image was both negative in valence and intrusive. The associated emotional-behavioral pattern could be described within a cognitive behavioral therapy framework. Eight patients also reported coping imagery.

**Conclusion:**

Negative pain-related cognitions in the form of intrusive mental imagery were reported by women with chronic pelvic pain. Targeting such imagery has led to interesting treatment innovation in the emotional disorders. Thus, imagery, hitherto neglected in pain phenomenology, could provide a novel target for cognitive behavioral therapy in chronic pain. These exciting yet preliminary results require replication and extension in a broader population of patients with chronic pain.

## Introduction

Pain suffering can be maintained or enhanced by negative cognitions. Therefore, exploring patients' cognitions about their pain is not only important for our understanding of their experience, but also to identify potentially unhelpful thoughts involved in symptom maintenance [1]. These could then be addressed through cognitive behavioral therapy (CBT).

Hitherto, most assessments of negative pain-related cognitions have focused on verbal thought processes (e.g., [2,3]). In contrast, spontaneous negative cognitions about pain in the form of mental images have rarely been explored in clinical populations. Mental images are cognitions with sensory-perceptual qualities, as opposed to thoughts, that is, thinking in words. These sensory qualities, while often visual, can involve any modality such as smell, touch, taste, and hearing. No specific measure has been developed to assess mental images in patients suffering from pain and we know little about their occurrence.

Very few studies have specifically investigated spontaneous mental imagery linked to pain symptomatology. Chaves and Brown [4] interviewed 75 patients about verbal thoughts and imagery experienced during acute dental pain: 15% of the patients experienced mental imagery either as a coping strategy, or as a form of catastrophizing. A postal questionnaire study of 83 patients with chronic pain concluded that 24% of respondents reported images linked to their pain [5]. In this study, the patients reporting imagery were more anxious and depressed than those who did not. A third study investigated images associated with chronic pain in patients suffering from irritable bowel syndrome, before and after hypnotherapy [6]. Out of 109 patients, 48.6% reported a mental image of their pain. The report of an image was associated with anxiety symptoms, but not with depression, quality of life, or symptom severity. In summary, the little clinical research to date suggests that both negative and positive cognitions about pain could take the form of mental imagery and that negative imagery may be linked with emotional suffering [4–6].

Despite some studies describing the coping imagery experienced in the context of acute pain [4,7,8], little is yet known about the contents and use of coping cognitions in the form of images in chronic pain. In fact, in that context, widely employed scales such as the Coping Strategies Questionnaire do not make a distinction between verbal thoughts and mental images, hence not allowing these to be identified and studied separately [9].

There is a wider literature on hypnosis and therapeutic imagery, which describes the exploration of imagery for pain relief [10]. In this therapeutic approach, patients are brought to focus on an image of their pain while they are in a relaxed or hypnotic state and modifications to this image are suggested (e.g., [10,11]). However, the focus is more on the effects of creating or exploring and modifying an image representing pain, than on the description of the images, or their potential presence outside of these therapeutic sessions.

A body of work from psychology suggests that cognitions in the form of imagery could be of importance in chronic pain. Mental images are a form of cognition with a more powerful impact on both negative and positive emotion than words [12,13]. We know that mental imagery recruits similar brain regions as those involved in real perception [14]. Moreover, compared with verbal thought, imagery has a stronger impact on behavior, learning, memory, and beliefs (see [15] for a review). Having negative pain-related verbal cognitions has been shown to impact on chronic pain outcomes (e.g., [16]). Thus, negative images related to pain, compared to negative verbal thoughts, would be predicted to have a stronger impact on the pain experience, the emotions associated with it, the behavioral outcomes, and so forth. While this type of cognition was recognized in the early days of CBT [17], research on mental imagery has only recently expanded. It has now been shown to be an important process in a range of mood and anxiety disorders including post-traumatic stress disorder and depression [18–28]. Interestingly, chronic pain is often associated with depression and anxiety, and they seem to reinforce each other [29,30]. Hence, negative mental imagery, if it occurred, would be predicted to participate in a negative vicious cycle amplifying pain and negative emotions. To illustrate this, Clark's model of anxiety [31] was adapted in the current work, with an emphasis on the role of imagery [28].

Chronic pelvic pain is described as pain in the pelvis lasting for at least 6 months, not occurring exclusively with menstruation or intercourse and not associated with pregnancy [32]. It has an incidence comparable with that of back pain or asthma [33]. It has been demonstrated that the presence or absence of a causal diagnosis in this condition does not have an impact on psychological distress and that the levels of anxiety and depression are similar to other chronic pain populations [34]. Patients were therefore recruited from a specialized tertiary referral clinic treating and investigating chronic pelvic pain in women [32,33,35] at the Nuffield Department of Obstetrics and Gynaecology in Oxford, thereby providing a relatively homogenous patient group in terms of gender and pain location.

**Figure 1:**
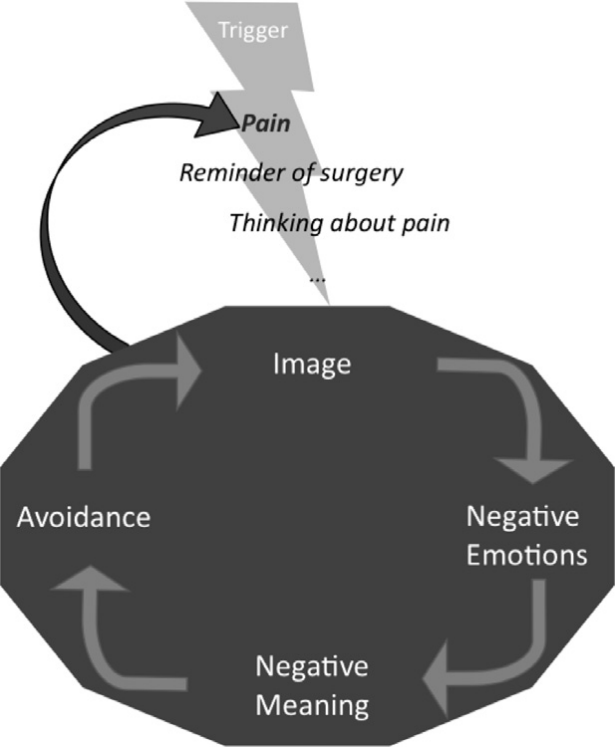
Cognitive behavioral therapy (CBT) formulation of the potential role of mental imagery in chronic pain. Triggers, such as pain or a reminder of the pain, evoke the image, which is linked to strong emotions, a negative interpretation (meaning), and avoidance. All this leads to a stronger/more negative experience of pain, and an increased likelihood of new occurrences of the image. The descriptions of patients' most relevant mental images in Table 1 are organized along this vicious cycle's structure.

The primary aim of this study was to assess if a group of women with chronic pelvic pain reported experiencing mental imagery related to pain, in addition to their verbal thoughts about pain. While our focus was on negative imagery as a potential treatment target, we were also curious as to whether any spontaneous positive (coping) imagery might occur. Furthermore, if this were the case, we sought to describe the nature of such imagery. A CBT framework was used by considering mental images as a form of cognition (thought with different properties than verbal ones) (Figure 1).

## Patients and Methods

### Patients

Patients treated at the Chronic Pelvic Pain Clinic of the Nuffield Department of Obstetrics and Gynaecology in Oxford were referred by their physician. Participants were reimbursed for their time plus transportation. The study was approved by the Oxfordshire Research Ethics Committee of the National Health Service (OXREC ref. 08/H0605/76).

Ten women (mean age 36.20 ± standard deviation [SD] 6.96 years) participated. Nine were Caucasian and one was Asian. Eight women had a diagnosis of endometriosis or adenomyosis, which they considered to explain part or all of their symptoms. Two women regarded their pain as unexplained. Self-reported somatic comorbidities were: rheumatoid arthritis (N = 1), fibromyalgia (N = 1), irritable bowel syndrome (N = 2), chronic shoulder pain following a traumatic injury (N = 1), and polycystic ovary syndrome (N = 1). Most reported abdominal surgery either for a caesarean (N = 3), for chronic pelvic pain-related investigations or treatments (N = 5), or for a gastric hernia (N = 1). A number of participants met criteria for the current axis I diagnoses: panic disorder (N = 3), depressive episode (N = 2), specific phobia (N = 1). History of comorbid lifetime disorders was as follows: past depression (N = 2), panic disorder (N = 2), bulimia (N = 1). None met diagnostic criteria for posttraumatic stress disorder.

### Measures

#### Cognitions about Pain Interview

The cognitions about pain interview was based on previous mental imagery studies [21,36–38]. Verbal thoughts and mental images were first defined with everyday examples and then explored separately, in a pseudo-randomized order across participants. For the purpose of the current article, the mental imagery interview is described in detail; the interview about verbal thoughts followed the same structure.

Participants were first asked an open-ended question: “We would like to know more about what you think when you are in pain. Can you think of any images that pop into your mind at that time?” Then they were asked whether they had experienced any image in any of 10 predetermined categories (e.g., “a time when your body was hurt,”“the pain as a thing or an object”). The participant gave a short description for each reported image. Care was taken to exclude descriptors used to communicate the nature of the pain to others but which were not spontaneous cognitions the patient would actually experience when in pain. Hence, if a metaphor was reported (e.g., “It feels like a fire”), participants were asked if it was a mental image or a descriptor. Responses in the category of “an image that makes you feel safe or better,” plus any other image described as positive, were computed as “coping” images.

Participants were then asked to choose the “most significant” mental image among those they had identified (i.e., the one they felt was most relevant to their pain, or the one they had most often when in pain). The most significant image was then explored in detail for content (“What is the image of?”), triggers (“Do you know any triggers for it?”), the type of emotion associated with it, the meaning it had for them (“What does it mean to you?”), the activities impaired by it (“Does it make you want to avoid certain activities? Which activities?”) as well as any behavior increased by it (“What does it make you want to do?”).

The most significant image was then rated on bidirectional 10 cm visual analogue scales (VAS) for the emotional valence (ranging from very negative, −10, to neutral, 0, to very positive, +10), and for the emotional impact (“How does it make you feel?”, from very much worse, −10, to neutral, 0, to very much better +10). In addition, unidirectional 10 cm VAS (ranging from 0, not at all, to 10, extremely or always, depending on the question) assessed both the cognitive (wishes for the image not to be there and worry) and the behavioral avoidance associated with the image as well as any behaviors it increased.

The corresponding data was collected on verbal thoughts in order not to bias the responses toward one type of cognition.

#### Standardized Questionnaires and Interviews

These measures were used to characterize the population; however, they were not main outcomes of the study.

*The Brief Pain Inventory short form (BPI)*[39] is a self-rating scale assessing pain severity and interference. It comprises a body map, on which the patient is expected to draw the localization of the pain. This map has been adapted for the gynecological pain context (http://www.pelvicpain.org/resources/handpform.aspx). A rating for the worst and least pain over the last 24 hours (scales from 0–10) is given. The interference in different fields is rated on scales from 0 to 10. Given the specificity of chronic pelvic pain, additional items were created for “interference with sexual relationship(s)” and “relations with other people.” A mean of the different interference scales is computed.

*The Pain Catastrophizing Scale (PCS)*[2] assesses verbal negative pain-related cognitions; it is a widely used scale that has shown predictive value in clinical populations [40].

*The Spontaneous Use of Imagery Scale (SUIS)*[41] is a self-report measure assessing the spontaneous use of mental imagery in everyday life.

*The Hospital Anxiety and Depression Score (HADS)*[42] is a self-report measure that assesses anxiety and depression in patients suffering from medical conditions.

*The Mini-International Neuropsychiatric Interview (M.I.N.I.)*[43] is an interview that allows assessment of the presence or absence of a psychiatric condition, along the *Diagnostic and Statistical Manual of Mental Disorders* (DSM-IV) criteria.

### Procedure

Participants received the standardized questionnaires at home, which they had to complete and bring to the meeting. A physician (the first author) led each of the interviews (approximately 2 hours). They were audio-recorded for supervision purposes. After giving written informed consent, the participant was asked about her pain (duration, cause, course over time, and treatments). The “cognitions about pain interview” was then completed, followed by the M.I.N.I. interview. The participant was then debriefed, assessing if they needed advice on where to get help if they felt distressed. However, none of the women required such help.

## Results

### Patient Characteristics

The mean duration of pain was 10.5 years (range 3–20 years). The mean worst pain score on the BPI was 5.44 (SD = 2.65) (this included two patients whose pain had significantly decreased as effective treatment had been initiated); the mean BPI interference score was 6.94 (SD = 1.79), which would be considered severe [39]. The average catastrophizing score on the PCS was 27.56 (SD = 14.04), which is considered high [2]. The mean score on the SUIS was 2.97 (SD = 1.49), indicating a standard level of imagery ability [41]. The mean HADS scores were: 10.33 (SD = 4.39) for anxiety, 9.56 (SD = 4.64) for depression, and 19.89 (SD = 8.07) for the total score, suggesting the presence of both clinical anxiety and depression for some participants [42] (data incomplete for one participant).

### Experience of Cognitions Linked to Pain

#### Negative Imagery

All participants reported having mental images in addition to verbal thoughts linked to their pain. On average, patients reported 4.6 (SD = 1.43) different negative images. Each patient's “most significant” mental image content is described in Table 1, alongside the identified triggers, associated emotion, meaning, and avoidance behavior. For seven patients, the image gave a meaning to the pain, or was an allegory of what pain did to them, while for three patients, the image was a memory of an actual event. These memories seemed to be linked to the consequences of the pain (e.g., patient feeling she is failing in her role of mother) or to the original onset of pain (e.g., operating room lights). While some of the images contained different sensory modalities (e.g., auditory for participant 5, somatosensory for participant 10), all had some visual elements.

**Table 1:** Description of patients' self-identified most significant image, with associated triggers, affect, meaning, and avoidance pattern

N°	Triggers	Content of Image	Associated Affect	Associated Meaning	Avoidance/Functional Impact
1.	Pain, sex, sleep.	I am being raped.	Repulsed, guilty, frightened, sick	A metaphor for the loss of control due to the pain.	Avoidance of triggers. I want to be a little girl, be looked after.
2.	Pain, thinking and talking about pain.	Malicious demons play around my pelvis, pushing, pulling and scratching.	Bitterly amused	Pain is malicious, out to get me.	Avoidance of physical activity.
3.	Pain, talking about pain.	Bright operating room lights.	Terrorized	I'm going to die.	Avoidance by filling mind with a substitution image (see text).
4.	Pain, anxiety.	I see myself as a small curled up figure in a red pit, grasping upwards.	Sad, helpless, anxious, tense	Something is wrong with me, and I cannot fix it.	Avoidance of many activities. I want to curl up and die.
5.	Pain, babies.	In the hospital room, I see and hear being told I need a caesarean.	Helpless and anxious	Could this difficult delivery explain the pain?	I cannot avoid the triggers.
6.	Pain, reminders of surgery or of menstruation, exercise.	A gaping hole in my abdomen, where my uterus and ovaries were. It is much bigger than it can be in reality and impenetrable.	Always sad, and at times angry or hopeful	This hole is keeping the space open for my organs to come back. I have not accepted my surgery.	Avoidance of triggers, restricting shopping and social interactions.
7.	Pain.	A little man jumping on my lower abdomen. He is the pain.	Helpless	The pain is winning.	Avoidance of many activities. Avoidance by filling mind with image of a number.
8.	Pain, social gatherings.	My children are crying.	Panicky, anxious and guilty	I am not giving my children a nice childhood. I am disappointing them.	Avoidance of triggers and activities with children.
9.	Pain.	Different things that could be happening inside me (e.g., my intestine is tearing or unzipping, there are ulcers in the bowel, my fallopian tubes are twisting).	Panicky while pain increases, then fed up.	They might have missed something. My pain is too important to be explained by my diagnosis.	Avoidance of many activities.
10.	Pain, movement.	There's a heavy, grey lead ball where it hurts. The weight is surprising, given its size. It's dragging down, as if it was going to fall out of me, taking my insides out.	Disgusted, horrified, revulsion	Something is wrong. This gives a meaning to the pain.	It brings up all my worries. I want to curl up in bed.

The most significant image had a negative emotional valence for all participants (M = −7.18, SD = 3.27). This image made the participants feel worse (M = −7.33, SD = 3.97); they wished that the image were not there (M = 5.73, SD = 4.29); they associated the image with worry (M = 6.24, SD = 3.10) and avoidance of activities (M = 7.90, SD = 3.00); the image was reported to increase pain-related behaviors (M = 7.34, SD = 3.00).

Nine participants described cognitive or behavioral avoidance linked to their most significant image (Table 1). Interestingly, for two participants, this took the form of a substitution mental imagery: for example, patient 3 described how bright operating lights were replaced in her mind's eye by an image of a black soft cube, which she then tried to make smaller. Furthermore, seven participants described avoidance of situations that were triggers of the image (such as reminders of a surgery, anxiety or potentially pain-increasing activities).

#### Coping Imagery

Interestingly, eight participants reported having one or more “coping” images when in pain (mean = 1.20, SD 0.92). For example, patient 1 described three spontaneous coping images: “I put the pain into a box, but this is difficult as no box is big enough.”; “I imagine that I grind an analgesic drug on my body, rubbing it in, or I imagine that I get an injection of a painkiller.”; and “I see my husband. He gives me support.” These examples summarize the coping imagery described by the participants, which one could describe in three categories (post-hoc): “allegoric treatment of an object symbolizing pain,”“imaginary treatment applied to the body,” and “supportive person.”

#### Verbal Thoughts

For eight participants, the most significant verbal thoughts were reported as being directly linked with a mental image. For example, for participant 5, the most significant verbal thought was “I am not being listened to,” and it was associated with an image of an operation she had undergone.

## Discussion

Interviews were performed on a small sample of patients (N = 10) with one specific chronic pain syndrome (chronic pelvic pain). This exploratory study represents a first step in investigating a neglected yet potentially important form of cognition in pain—mental imagery. On average, the group had mild pain intensity but severe interference due to pain, high catastrophizing, and typical trait imagery ability. That all patients reported negative mental imagery associated with their symptoms, provides encouraging support for the importance of this rarely considered type of thought process in pain suffering. All interview responses were consistent with a classic CBT vicious cycle, whereby the negative imagery could amplify pain suffering (see Figure 1). The proposed vicious cycle is based on previous formulations using imagery [28]. While this is a hypothetical model, it is interesting to note that in an experimental context, images can contribute to fear conditioning [44]. Furthermore, abnormal memory formation or extinction has been proposed to contribute to the pathological process underlying the chronification of pain [45]. Images of difficult memories associated with pain were common among the cognitions described by the patients in this study. This suggests that mental imagery could be a richer source of enquiry about patient's experience of pain, including their beliefs and fears, than purely verbal report.

Interpreting these findings in a CBT framework could be helpful for developing new therapies; however, at this stage of research, we remain open to alternative explanatory models. Further research should confirm that these images have an emotional impact and are not merely visual representations of the pain facilitating its acknowledgment. In fact, all mental images contained visual elements, which is interestingly similar to findings in post-traumatic stress disorder [46], but other sensory modalities were also described.

There has been some indication of imagery-based pain-related negative cognitions in previous research [4–6], however, with lower rates of occurrence. The method of the present study differed from the previous work, as a face-to-face interview focusing on the different forms of cognitions was performed, specifically in patients with chronic pain. A psychology-trained physician who was not in charge of the patients' treatment conducted the interview. This might have helped the patients to disclose their cognitions. Patients with chronic pelvic pain often do not feel that physicians are taking their symptoms seriously [35]. Therefore, they might be worried such cognitions could affect how their doctor perceives them. Moreover, the sample presented here is very small and relatively homogenous. A similar approach has been taken in mapping out novel symptomatalogy in other clinical groups (e.g., suicidal flashforwards and agoraphobia; [21,37]). Thus, following a wider research tradition in other disorders, this pilot interview study should be replicated in different pain conditions and larger populations of patients.

Our hypothesis focused on negative pain-related cognitions, and the interview was setup to assess this. Interestingly, while all images considered as most relevant by the patients were of negative valence, some patients also reported spontaneous coping imagery. Similar findings have been previously described in the context of acute dental pain [4] and in experimental pain settings [7,8]. Such coping strategies can provide control over pain in the experimental context [47]; however, it is not yet known if they have the same effect in chronic pain, or if they are dysfunctional and act as avoidance. This form of coping cognition warrants further exploration in the future.

In conclusion, this exploratory study confirmed that cognitions about chronic pain could indeed take the form of mental imagery (as suggested previously). Furthermore, the idiosyncratic contents of these intrusive images were described. The study is limited by its small sample size, yet it is able to conclude that spontaneous mental images do occur in chronic pain patients. Idiosyncratic intrusive negative imagery has hitherto been neglected in pain treatment. Yet, as in other disorders, such negative (pain related) cognitions may fit a classic CBT vicious cycle, involving negative interpretations, emotions, and avoidance. If so, chronic pain patients' own unique spontaneous mental imagery could represent a useful and specific target for therapy, as in other disorders [48]. However, the causal status and malleability of such imagery remains to be explored. The potential for treatment innovation targeting such imagery is of particular importance for future research given the poor relief provided to chronic pain patients by currently available treatment strategies and the frequent comorbidity with emotional disorders. This research offers a promising first step in this direction.
